# Numerical design of magnetic-field-gradient-guided branch targeting of Au/Ni–SiO_2_ Janus microrobots in a bifurcating microchannel

**DOI:** 10.1039/d6ra08407a

**Published:** 2026-10-13

**Authors:** Junbo Yang, Meijuan Li, Yonghui Yang, Xue-Bo Chen

**Affiliations:** a School of Electronic and Information Engineering, University of Science and Technology Liaoning Anshan 114051 China xuebochen@126.com

## Abstract

The controllable branch guidance of magnetic Janus microrobots in bifurcating microchannels is governed by the coupled effects of background advection, magnetophoretic migration and particle structure. Here, a three-dimensional numerical framework was developed to investigate the magnetically controlled transport of Au/Ni–SiO_2_ Janus microrobots in an idealised low-Reynolds-number bifurcating microchannel. An equivalent three-axis Helmholtz–Maxwell coil model was used to reconstruct an oblique magnetic-field gradient directed towards the target branch, and the resulting electromagnetic field was coupled with particle tracking. Over 0.5–7.0 T m^−1^, the reconstructed gradients closely reproduced the prescribed values, whilst the magnetic flux density remained above the adopted near-saturation criterion for Ni. For the baseline microrobot with a 120 nm Ni layer, the target-branch transport efficiency increased from 42.67% at 1.5 T m^−1^ to 100% at 7.0 T m^−1^. Increasing the inlet velocity from 0.25 to 1.50 mm s^−1^ shifted the guidance response towards higher gradients, reflecting stronger competition between axial advection and lateral magnetophoretic migration. Increasing the Ni-layer thickness from 150 to 390 nm raised the magnetic Ni volume fraction from 8.45% to 20.74% and reduced the minimum tested gradient for complete target-branch transport from 5.5 to 2.5 T m^−1^, a reduction of approximately 54.5%. Mechanistic analysis showed that the increase in magnetic moment substantially exceeded the increase in hydrodynamic resistance, and the transport data for different Ni-layer thicknesses were largely represented by a common response using a dimensionless magnetic-guidance parameter. These results provide a quantitative basis for co-optimising microrobot magnetic structure, background flow and external magnetic actuation.

## Abstract 2

Coupled optimisation of magnetic actuation and Ni-layer structure enables magnetic-field-gradient-guided targeting of Au/Ni–SiO_2_ Janus microrobots, reducing the gradient required for efficient transport.

## Graphical abstract

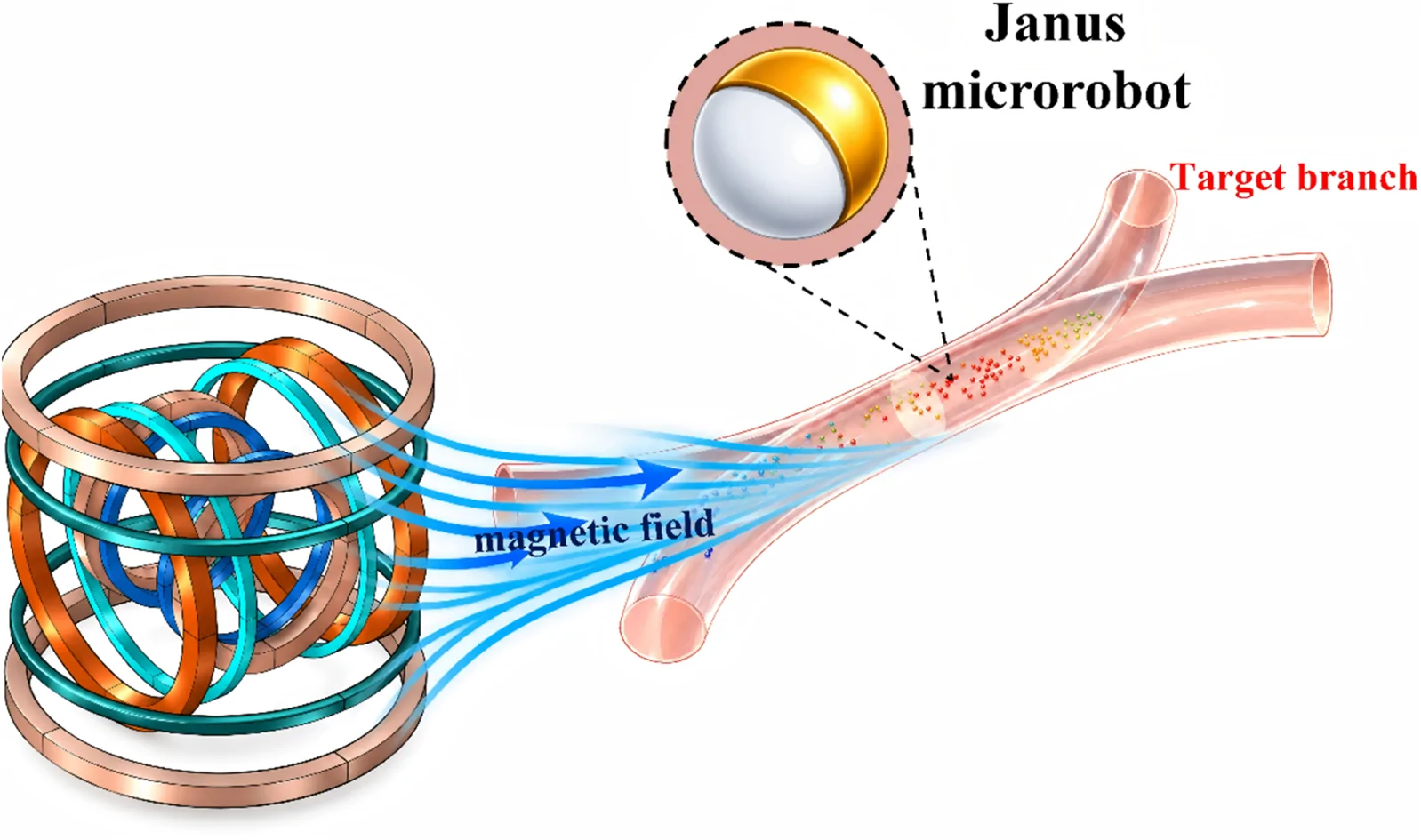

## 1 Introduction

Micro- and nanorobots have considerable potential for applications in targeted delivery, microfluidic manipulation, biological sample processing, and localised sensing, owing to their capabilities for active motion, programmable control, and microscale material transport within confined environments.^1–3^ With continuing advances in micro- and nanofabrication, functional materials, and external-field actuation technologies, achieving stable, predictable, and controllable microrobot motion in complex fluidic environments has become an important research challenge.^4,5^ Among external actuation modalities based on magnetic, optical, acoustic, and electric fields, magnetic actuation offers several advantages, including non-contact operation, remote controllability, no requirement for an onboard energy source, and penetration through most non-magnetic media, making it particularly suitable for microscale manipulation in enclosed or confined liquid environments.^6–8^ At the microscale, particle motion generally occurs under low-Reynolds-number conditions, where inertial effects are relatively weak and viscous effects dominate. Consequently, the transport of microrobots is governed not solely by externally applied magnetic forces, but by the combined effects of magnetic actuation, viscous drag, and background advection.^9^ A multiphysics model capable of resolving the externally applied magnetic field, local flow field, and particle motion within a unified framework is therefore essential for understanding the controlled transport of magnetic microrobots and for optimising their actuation parameters.

Spatial gradients in a non-uniform magnetic field can exert translational magnetic forces on magnetically responsive particles or microrobots, causing them to deviate from their background streamlines and undergo controllable magnetophoretic migration. On the basis of this mechanism, magnetophoresis has been widely employed for magnetic-particle transport, cell sorting, target capture, and sample enrichment in continuous-flow microfluidic systems.^10,11^ In bifurcating channels, where the flow divides into separate downstream branches, particles must acquire sufficient lateral displacement within a finite residence time to migrate from their original background streamlines towards the target branch.

Therefore, compared with magnetic-field magnitude alone, the spatial magnetic-field gradient and the resulting lateral magnetophoretic migration are more directly relevant to branch selection.

Recent numerical studies of continuous-flow magnetophoresis have further shown that the representation of the flow field, the spatial distribution of the magnetic field, and the particle-dynamics model can all affect predictions of magnetic-particle transport and outlet partitioning.^12–14^ Incorporating magnetic-field-gradient reconstruction, low-Reynolds-number flow, and particle-trajectory tracking into a unified numerical framework can therefore facilitate the establishment of quantitative relationships between external actuation parameters and the resulting branch-transport performance.

Janus particles, which contain spatially distinct regions with different physical or chemical properties within a single particle, provide a versatile platform for the integrated design of magnetic response, surface functionalisation, and microscale motion.^15–17^ In particular, the response of magnetic Janus particles to externally applied magnetic fields can be tailored by modifying the composition, spatial distribution, and structural configuration of their magnetic constituents. Such particles have been employed in magnetically controlled transport, target detection, and biomedical microscale manipulation.^16–20^ For SiO_2_-based Janus systems, experimental studies have demonstrated the fabrication of magnetically responsive Janus microparticles by depositing a Ni magnetic layer, or a Ni/Au metallic hemishell, on one side of SiO_2_ microspheres. In these structures, the Ni layer provides the primary magnetic response, whereas the outer Au layer offers a stable metallic interface for subsequent surface functionalisation.^18–20^ These studies provide an experimental materials basis for the structural design of Au/Ni–SiO_2_ Janus microrobots.

Importantly, for such multilayer composite particles, increasing the Ni-layer thickness not only increases the amount of magnetic material but also alters the total particle volume, mass, effective density, and hydrodynamic size. Increasing the Ni-layer thickness may therefore enhance the magnetophoretic driving force by increasing the volume of magnetic material, while simultaneously modifying the hydrodynamic drag and effective gravitational force. Consequently, an increase in magnetic-material content cannot be assumed to produce a proportional improvement in particle-transport performance; instead, the effective magnetically controlled transport behaviour should be evaluated by considering the coupled effects of particle structural parameters and the externally applied magnetic field. A mechanistic assessment of the relative changes in magnetic response and hydrodynamic resistance is therefore required to distinguish the principal contribution of the magnetic layer from the other structural changes introduced by increasing its thickness.

Magnetic microrobot transport through bifurcating microchannels and other confined-flow environments has been investigated previously. Existing studies have shown that background flow velocity, robot size, initial position, branch geometry, and magnetic-field conditions can all affect trajectory stability and branch-navigation performance.^21,22^ In particular, under continuous background flow, the externally applied magnetic actuation must generate sufficient lateral migration within the finite time available before the particle reaches, or while it traverses, the bifurcation region, thereby shifting its position relative to the streamline separatrix. Magnetic-field-gradient-controlled navigation through bifurcations is therefore not, in itself, an unexplored problem. However, previous studies have focused primarily on the effects of background flow, overall robot size, bifurcation geometry, or navigation strategy on the magnetic actuation requirements.^23,24^ By comparison, for multilayer magnetic Janus structures with an established experimental materials basis, there remains a lack of systematic quantitative understanding of how magnetic-layer structure interacts with the externally applied magnetic-field gradient and background advection to determine branch-transport performance. In particular, it remains unclear whether structural optimisation of the magnetic component can reduce the external actuation required to achieve high-efficiency or complete target-branch transport, and which of the coupled changes in magnetic and hydrodynamic properties primarily governs this improvement.This issue is particularly relevant to Au/Ni–SiO_2_ Janus microrobots because increasing the Ni-layer thickness simultaneously modifies their magnetic response and hydrodynamically relevant parameters, thereby coupling particle structure to the magnetic-field gradient. A unified parametric framework capable of relating particle structure, external magnetic actuation, background flow and outlet partitioning is therefore required, together with a mechanistic descriptor that can account for the competition between magnetophoretic driving, viscous resistance and advective transport.

On this basis, the present study establishes a numerical framework for the magnetically controlled transport of Au/Ni–SiO_2_ Janus microrobots through a three-dimensional bifurcating microchannel. For the translational-dynamics calculations, the actual Janus structure is represented by an equivalent rigid sphere with the same total volume and mass, while retaining the contribution of the actual Ni magnetic component to the magnetophoretic driving force. This treatment reduces the geometric complexity of the particle model whilst preserving the correspondence between the particle mass and magnetic-response parameters and those of the actual structure. For magnetic actuation, an equivalent three-axis Helmholtz–Maxwell coil model is employed, and an oblique magnetic-field gradient directed towards the target branch is reconstructed within the local working region through the linear superposition of the unit-current responses of the individual coil pairs. The reconstructed magnetic-field magnitude and gradient are evaluated consistently from the combined field, and the local magnetic flux density is further examined against the adopted near-saturation criterion for Ni before the reconstructed field is applied to particle tracking. The low-Reynolds-number background flow, Stokes viscous drag, magnetophoretic driving force, and effective gravitational force are subsequently coupled within an Euler–Lagrange framework to enable quantitative prediction of particle trajectories and outlet partitioning. The reconstruction performance of prescribed magnetic-field gradients over the investigated range of 0.5–7.0 T m^−1^ is first evaluated. The influence of magnetic-field-gradient magnitude on the target-branch transport of the baseline Janus microrobot is then quantified, followed by a systematic assessment of inlet flow velocity to elucidate the competition between lateral magnetophoretic migration and background advection. The Ni-layer thickness is subsequently varied to determine how changes in magnetic-material content and the accompanying structural properties modify the magnetic-field conditions required for target-branch transport.

To clarify the physical origin of the Ni-layer effect, the associated changes in magnetic moment, particle mass, effective density, and hydrodynamic resistance are further compared, and the particle-transport results are expressed using a dimensionless magnetic-guidance parameter that characterises the competition amongst magnetophoretic driving, viscous resistance, and background advection. Target-branch transport efficiency and the minimum tested gradient at which 100% target-branch transport is first achieved within the present discrete parameter sweep are used as quantitative performance metrics.

The principal contribution of this work is therefore not the demonstration of magnetic branch navigation itself, but the establishment of a coupled numerical framework that quantitatively relates magnetic-layer structure, external magnetic-field gradient, and background advection to branch-selection performance.

## 2 Numerical modelling and simulation methods

### 2.1 Janus microrobot structure and equivalent particle parameters

Spherical magnetic Au/Ni–SiO_2_ Janus microrobots were considered in this study. Previous studies have reported the fabrication of SiO_2_-based magnetic Janus microparticles with metallic thin-film coatings, including the deposition of a magnetic Ni layer on one side of a SiO_2_ microsphere and the subsequent formation of a Ni/Au metallic hemishell.^25^

The Ni layer provides the magnetic response to the externally applied field, whereas the outer Au layer serves as a metallic interface for subsequent surface functionalisation. As illustrated in Fig. 1, the SiO_2_ core constitutes the primary supporting structure of the microrobot, the Ni layer provides the magnetic response, and the Au layer forms the outer metallic interface of the particle.

**Fig. 1:**
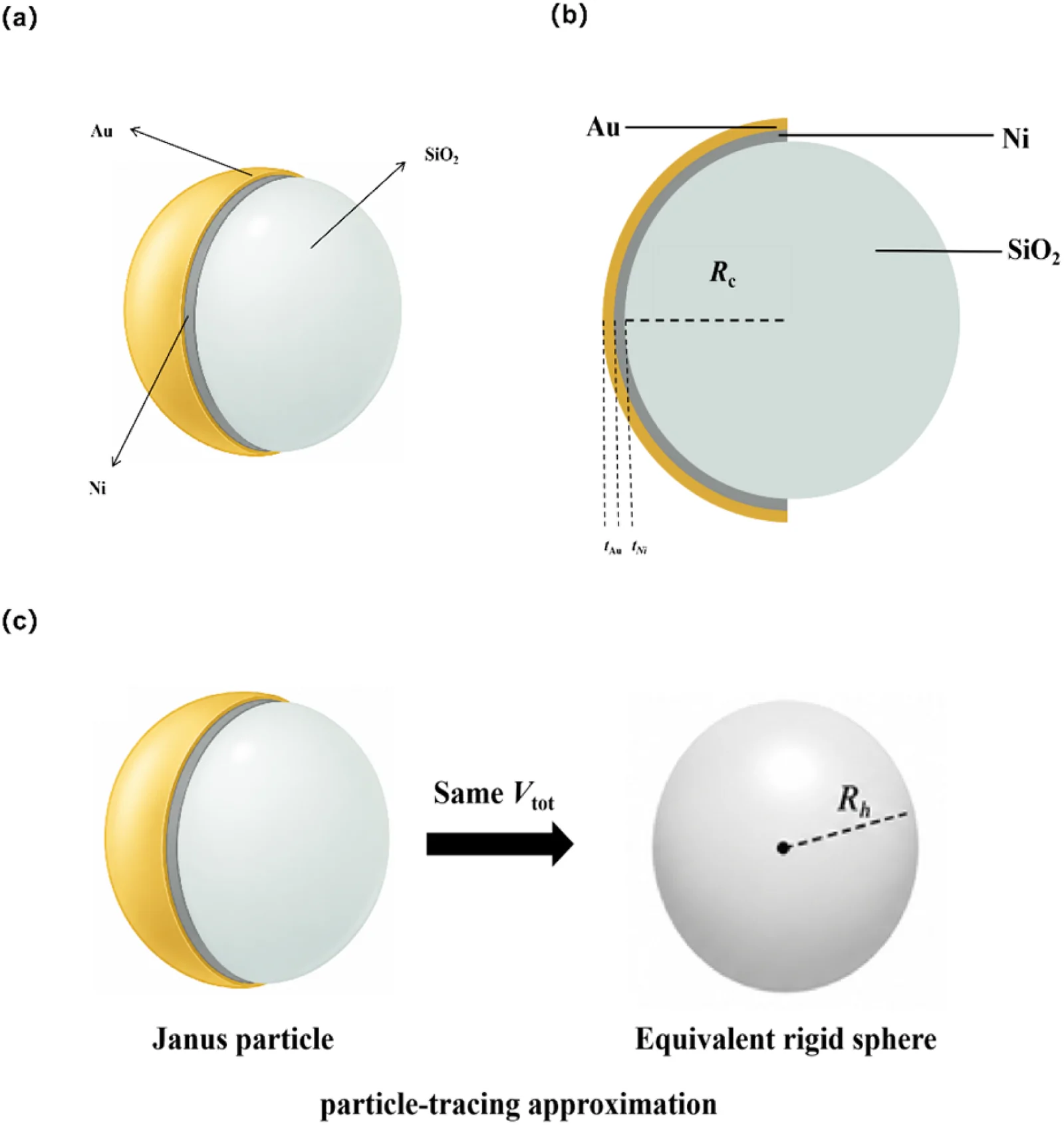
Structure of the Au/Ni–SiO_2_ Janus microrobot and its equivalent-particle representation. (a) Schematic three-dimensional structure of the Au/Ni–SiO_2_ Janus particle. (b) Definition of the geometric parameters of the SiO_2_ core, Ni hemispherical shell, and Au hemispherical shell. (c) Equal-volume, equal-mass rigid-sphere approximation used for particle tracking.

Because the present study focuses on the translational transport and branch selection of Janus microrobots in a confined flow field, rather than on the orientational dynamics arising from their asymmetric geometry, the actual Janus structure was represented by a rigid spherical particle with the same total volume and total mass to reduce the complexity of the particle-tracking model. This equivalent representation was used only for the hydrodynamic description of particle translation; the magnetic response of the particle remained determined by the volume of the actual Ni magnetic component. The model therefore retains the principal effects of variations in particle mass, characteristic hydrodynamic size, and magnetic-material content with structural parameters, whilst the rotational dynamics and orientation-dependent drag associated with the asymmetric Janus geometry are not explicitly resolved.

Let *R*_c_ denote the radius of the SiO_2_ core, *t*_Ni_ the Ni-layer thickness, and *t*_Au_ the Au-layer thickness. For the baseline Au/Ni–SiO_2_ Janus microrobot, the SiO_2_-core radius was set to *R*_c_ = 2.5 µm, the Au-layer thickness was fixed at *t*_Au_ = 50 nm, and the Ni-layer thickness was set to *t*_Ni_ = 120 nm. In the subsequent parametric analysis of the Ni-layer structure, the SiO_2_-core size and Au-layer thickness were held constant, whilst only the Ni-layer thickness was varied to investigate the effect of the magnetic-layer structure on the magnetically controlled transport performance of the microrobot.

According to the geometry of the hemispherical shells, the volumes of the three constituent components are given by:1
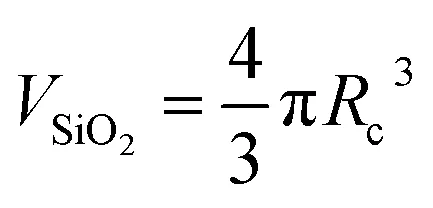
2
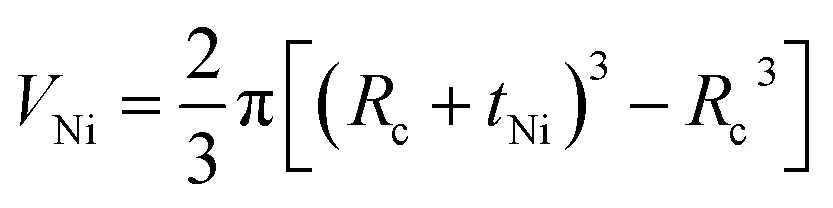
3
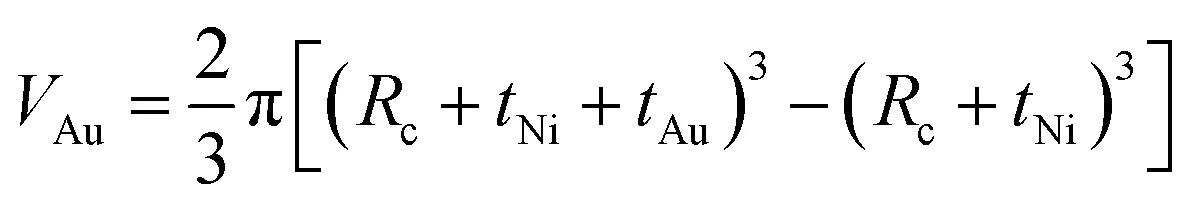


The total volume of the Janus microrobot is therefore

4 *V*_tot_ = *V*_SiO_2__ + *V*_Ni_ + *V*_Au_

Accounting for the contribution of each material component to the particle mass, the effective particle density was calculated using volume weighting:5
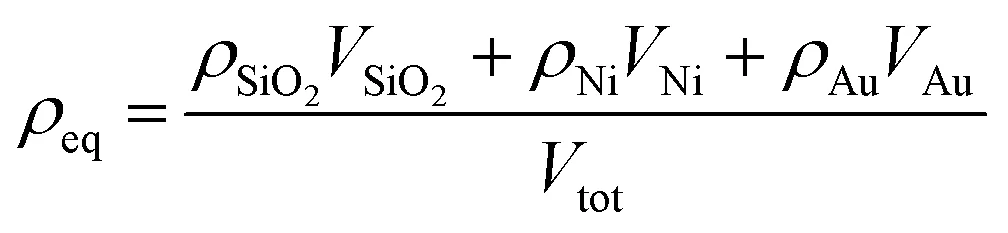


The material densities of SiO_2_, Ni, and Au were taken as 2200, 8908, and 19 300 kg m^−3^, respectively, with bulk–material properties adopted as a continuum approximation.^26^ The corresponding effective particle mass is

6 *m*_p_ = *ρ*_eq_*V*_tot_

Because the present study focuses primarily on translational transport and does not explicitly resolve the orientation-dependent hydrodynamic drag arising from the asymmetric Janus geometry, an equivalent sphere having the same total volume as the actual composite particle was defined. Its effective hydrodynamic radius is expressed as7
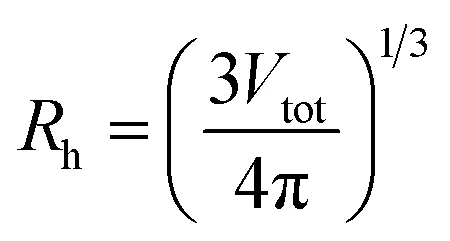


The volume fraction of the magnetic Ni component within the composite particle is defined as8
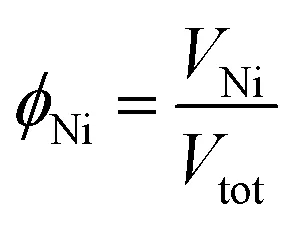
Here, *ϕ*_Ni_ characterises the relative content of magnetic material within the composite particle. It should be emphasised that increasing the Ni-layer thickness changes not only the magnetic-material content but also the total particle volume, effective density, mass, and effective hydrodynamic radius. Accordingly, in the subsequent structural-parameter analysis, *ϕ*_Ni_ should be interpreted as an integrated descriptor of variations in the Ni-layer structure rather than, in the absence of a controlled-variable analysis, as a unique independent parameter governing the magnetic response.

For the baseline Au/Ni–SiO_2_ Janus structure with a Ni-layer thickness of 120 nm, the calculated value of *ϕ*_Ni_ was 6.808%. With the SiO_2_-core size and Au-layer thickness held constant, increasing the Ni-layer thickness from approximately 150 to 390 nm increased *ϕ*_Ni_ from 8.45% to 20.74%.This structural-parameter range is comparable to experimentally reported dimensions for magnetic Janus microrobots.^20,27,28^ The effect of increasing the Ni magnetic-layer thickness on the magnetically controlled transport behaviour of the Janus microrobot can therefore be systematically investigated without altering the geometric parameters of the SiO_2_ core or Au layer.

### 2.2. Bifurcating-channel model and flow boundary conditions

To investigate the branch-selection behaviour of Au/Ni–SiO_2_ Janus microrobots under confined low-Reynolds-number flow conditions, an idealised three-dimensional dual-inlet–dual-outlet bifurcating microchannel was constructed. As shown in Fig. 2, the model comprises two upstream inlets, a common main channel, and two downstream outlets. The channel has a cross-sectional diameter of 1.00 mm and a common-main-channel length of 3.70 mm, whilst the separation between the two indicated reference points is 7.00 mm. Each branch is oriented at 45° with respect to the corresponding coordinate axis.

**Fig. 2:**
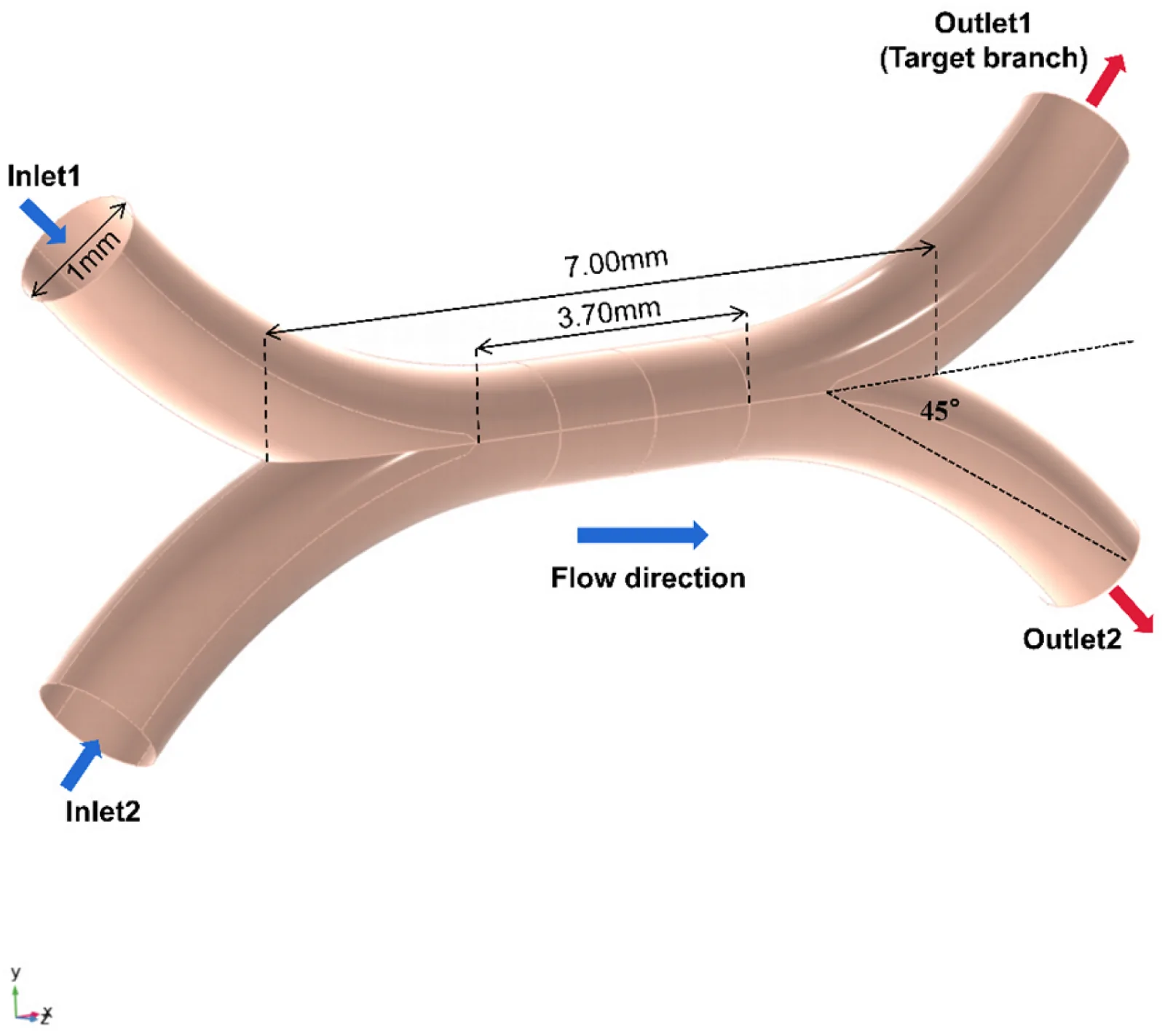
Geometry, flow direction, and outlet definitions of the idealised three-dimensional dual-inlet–dual-outlet bifurcating microchannel. Outlet 1 is defined as the target branch and outlet 2 as the non-target branch; the axial direction of the common main channel is defined as the (*x*)-direction.

To isolate the competition between background advection and lateral magnetophoretic migration, whole blood was approximated as an incompressible Newtonian fluid in the baseline flow model.

The fluid density and dynamic viscosity were set to 1060 kg m^−3^ and 0.004 Pa s, respectively; comparable parameter combinations have previously been employed in Newtonian approximations for computational fluid dynamics simulations of blood flow.^29,30^ A mean inlet velocity of 0.5 mm s^−1^ was prescribed at both inlets to establish a controlled low-Reynolds-number background flow.^21^ Taking the channel cross-sectional diameter of 1 mm as the characteristic length, the corresponding Reynolds number is approximately9
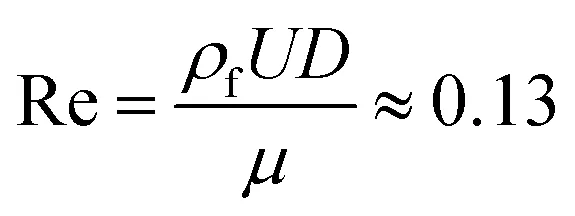


This value confirms that the flow lies within the low-Reynolds-number regime, in which viscous effects dominate. It should be noted that treating blood as a constant-viscosity Newtonian fluid constitutes a modelling simplification adopted to isolate and quantitatively examine the fundamental competition between background advective transport and lateral magnetophoretic migration. It does not imply that blood rheology in real microvessels can be adequately represented by a Newtonian model under all conditions. The present model does not account for vessel-wall elasticity, pulsatile flow, red-blood-cell-scale interactions, cell-particle collisions, biochemical interactions at the channel wall, or the non-Newtonian rheology of blood. The model should therefore be regarded as an idealised confined-flow representation designed to elucidate the fundamental behaviour of magnetically controlled branch selection, rather than as a complete reproduction of an *in vivo* vascular environment.

Under steady-state conditions, the continuous phase satisfies the incompressible continuity and Navier–Stokes equations:

10 ∇·**u** = 0

11 *ρ*_f_(**u**·∇)**u** = −∇*p* + *µ*∇^2^**u**

Outlet 1 was defined as the target branch and outlet 2 as the non-target branch, with a pressure boundary condition of zero imposed at both outlets. The axial direction of the common main channel was defined as the (*x*)-direction, whilst lateral branch deflection occurred in the (*x*–*y*) plane. The steady laminar velocity field was first solved under the prescribed boundary conditions and was subsequently used as the background continuous-phase velocity field in the Euler–Lagrange particle-tracking model. Au/Ni–SiO_2_ Janus microrobots were released from inlet 2 and transported downstream towards the bifurcation region by the background flow. The applied oblique magnetic-field gradient generated lateral magnetophoretic displacement during the finite residence time of the particles within the common main channel and bifurcation region. When this lateral displacement was sufficient for a particle to cross its original streamline or the separatrix defining branch allocation, its downstream outlet selection was altered. The numbers of particles ultimately entering outlets 1 and 2 were recorded using outlet counters and were used to calculate the target-branch transport fraction.

To ensure strict comparability among different magnetic-field conditions, all particle-tracking simulations employed identical channel geometry, inlet velocity, outlet boundary conditions, and particle-release protocols; only the magnetic-field or particle–structure parameters specified for each analysis were varied. This idealised bifurcating channel therefore provides a consistent numerical platform for quantitatively analysing the competition between magnetophoretic actuation and background advection under low-Reynolds-number conditions.

### 2.3. Magnetic-field-gradient reconstruction and equivalent coil actuation

Helmholtz–Maxwell combined-coil systems are widely used for the electromagnetic actuation of magnetic microrobots.^31–33^ Helmholtz coils are primarily employed to generate relatively uniform magnetic fields with controllable directions, whereas Maxwell coils are mainly used to generate non-uniform magnetic fields with spatial gradients. These fields provide the conditions required for the magnetisation and magnetic-moment response of magnetic materials and for magnetic-gradient actuation, respectively. To obtain magnetic fields and magnetic-field gradients with controllable directions and magnitudes within the working region of the bifurcating microchannel, a three-dimensional equivalent electromagnetic coil model comprising three pairs of Helmholtz coils and three pairs of Maxwell coils was established around the channel. As shown in Fig. 3, the three Helmholtz coil pairs and three Maxwell coil pairs are arranged along three mutually orthogonal coordinate axes.

**Fig. 3:**
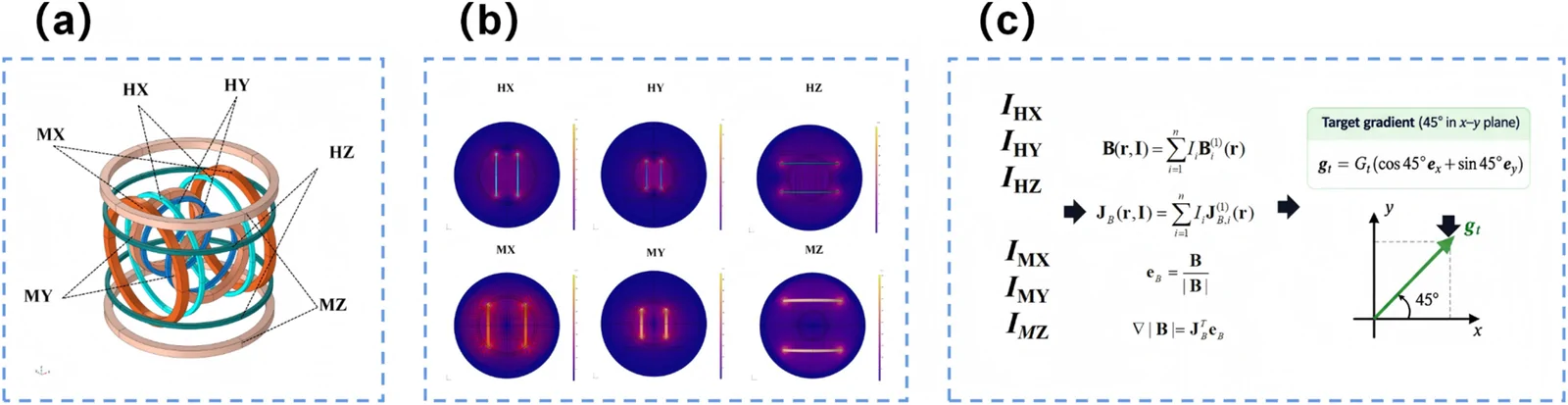
Three-dimensional combined-coil system and magnetic-field-gradient reconstruction procedure. (a) Three-axis Helmholtz–Maxwell coil assembly; (b) unit-current magnetic-field response of each coil set; (c) linear superposition based on the unit-current responses.

Under magnetostatic conditions, the magnetic-field response generated by a coil is approximately linear with respect to the driving current. Therefore, the unit-current-response linear-superposition approach was employed for magnetic-field reconstruction. First, the spatial magnetic-field responses generated by the six coil pairs, HX, HY, HZ, MX, MY and MZ, were calculated separately at a unit current of 1 A. The principle of electromagnetic linear superposition was then used to obtain the total magnetic field and its spatial gradient within the working region for any prescribed set of six coil currents. This approach avoids the need to resolve the complete electromagnetic-field model for every set of driving currents and provides a consistent basis for subsequent magnetic-field parameter sweeps and particle-tracking simulations.

For the *i* th coil pair, the magnetic flux density generated at a unit current of 1 A is defined as:

12 **B**_*i*_^(1)^(**r**)

The corresponding magnetic-field Jacobian is given by:

13 **J**_*B*,*i*_^(1)^(**r**) = ∇**B**_*i*_^(1)^(**r**)

For the coil-current vector

14 **I** = [*I*_HX_, *I*_HY_, *I*_HZ_, *I*_MX_, *I*_MY_, *I*_MZ_]^*T*^

The magnetic flux density and its Jacobian at an arbitrary position (*r*) can be expressed, according to linear superposition, as15
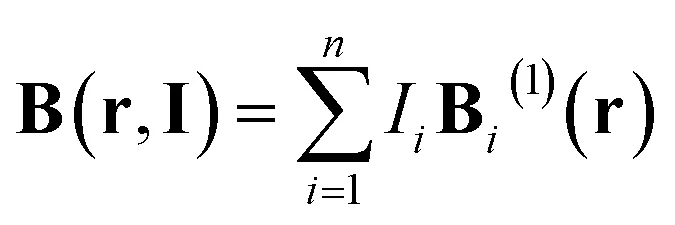
16
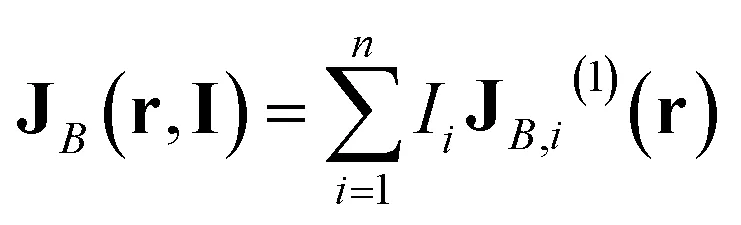


The unit vector in the local magnetic-field direction is defined as17
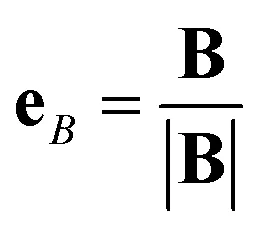


The spatial gradient of the magnetic-flux-density magnitude can then be calculated from

18 ∇|**B**| = **J**_B_^*T*^**e**_B_

The objective of the magnetic-field reconstruction was not to obtain a strictly uniform magnetic-field gradient throughout the entire computational domain, but rather to reconstruct a local magnetic-field gradient with a well-defined direction and tunable magnitude within the finite working region traversed by the Janus microrobots in the bifurcating-microchannel model. To deflect Janus microrobots released from inlet 2 towards target outlet 1, the prescribed magnetic-field gradient was oriented in the (*x*–*y*) plane at an angle of 45° to the main-channel axis. The target gradient vector was defined as

19 **g**_*t*_ = *G*_*t*_(cos 45°**e**_*x*_ + sin 45°**e**_*y*_

Here, *G*_*t*_ denotes the magnitude of the prescribed magnetic-field gradient.

A set of sampling points was selected within the working region, and a gradient-response matrix (*A*) was constructed from the local magnetic-field-gradient responses of the individual coils under unit current. For a prescribed target gradient, the coil driving currents were determined by minimising the discrepancy between the reconstructed and target gradients:20
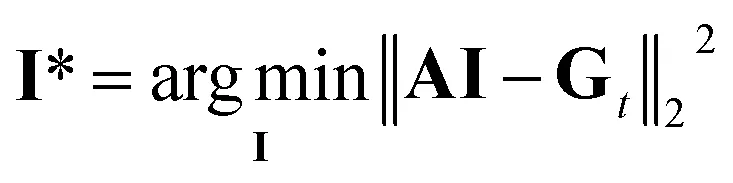


The corresponding least-squares solution is

21 I* = **A**^+^**G**_*t*_

where **A**^+^ denotes the Moore–Penrose pseudoinverse of the response matrix (*A*).

Substitution of the resulting coil-current vector (I*) into eqn (15) and (16) yields the reconstructed magnetic field and the spatial gradient of its magnitude within the working region. The reconstruction accuracy and spatial uniformity of the magnetic-field gradient were subsequently evaluated by comparing the mean, maximum, and minimum reconstructed gradients with the prescribed gradient (*G*_*t*_).

This procedure establishes a quantitative relationship between the prescribed magnetic-field gradient and the individual coil driving currents, whilst enabling continuous adjustment of the gradient magnitude with the overall spatial characteristics of the magnetic field remaining essentially unchanged. It therefore provides controlled magnetic conditions for the subsequent investigation of magnetic-field-gradient-dependent branch transport of Janus microrobots.

### 2.4 Janus microrobot dynamics and particle-tracking model

After obtaining the steady-state continuous-phase velocity field and spatial magnetic field, the transient transport of the Janus microrobots was solved within an Euler–Lagrange framework. Because the particle suspension considered in this study was dilute, individual microrobots were assumed to behave independently. Particle–particle collisions, magnetic dipole–dipole interactions, agglomeration, and the feedback of particle motion on the background flow field were neglected. A one-way coupling scheme was therefore employed between the continuous and particle phases.

The translational motion of a particle is governed by Newton's second law:22
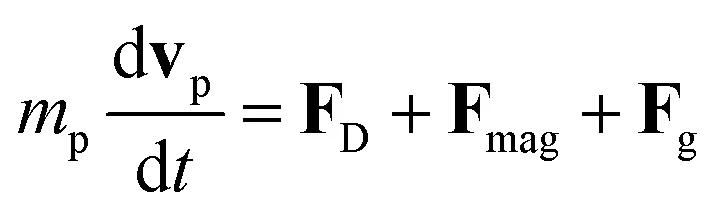
Here, (**v**_p_) is the particle velocity, whilst (**F**_D_), (**F**_mag_), and (**F**_g_) denote the viscous drag force, magnetic-field-gradient force, and effective gravitational force accounting for buoyancy, respectively.

Under low particle-Reynolds-number conditions, the hydrodynamic drag acting on the particle was described using the Stokes drag model:

23 **F**_D_ = 6π*µR*_h_(**u** − **v**_p_)

This expression assumes that the relative motion between the particle and the background fluid occurs in a viscous-drag-dominated regime. Because the actual Janus particle was represented by an equivalent sphere, the model does not explicitly account for orientation-dependent drag arising from the asymmetric Janus geometry, nor does it incorporate near-wall lubrication corrections or particle-surface-roughness effects.

For a magnetic particle whose magnetic moment can align with the local magnetic field, the translational force generated by a non-uniform magnetic field can be described through the interaction between the magnetic moment and the magnetic-field gradient. The magnetic-gradient force acting on the particle is generally expressed as

24 **F**_mag_ = ∇(**m**_eq_·**B**)

If the Ni magnetic moment is assumed to align rapidly with the local magnetic-field direction, then

25 *F*_mag_ = *V*_Ni_*M*_Ni_(|**B**|)**J**_B_^*T*^**e**_B_

Under the further assumption of magnetic saturation,

26 **F**_mag_ = *M*_s_*V*_Ni_∇|**B**|

Here, (*M*_s_) is the saturation magnetisation of Ni. Under the maximum-magnetic-response approximation, (*M*_s_) was taken as 4.90 × 10^5^ Am^−1^, consistent with a typical room-temperature saturation magnetisation of bulk Ni.^34^ It should be emphasised that eqn (26) represents a saturation-magnetisation, or maximum-magnetic-response, approximation and is applicable only when the Ni magnetic layer is close to magnetic saturation and its effective magnetic moment can respond rapidly to the local magnetic-field direction.

The effective gravitational force acting on the particle is determined jointly by the particle weight and fluid buoyancy:

27 **F**_g_ = (*ρ*_eq_ − *ρ*_f_)*V*_tot_**g**

This term describes the tendency of the particle to sediment or rise as a consequence of the density difference between the particle and the surrounding fluid. As the Ni-layer thickness changes, the total volume, effective radius, effective density, and mass of the Janus microrobot also change. Consequently, the Ni-layer structure affects the magnetophoretic driving force, viscous drag, and effective gravitational force simultaneously, rather than modifying the magnetic force alone.

Particle contact with the channel wall was treated using a specular-reflection boundary condition. When a particle reaches a solid wall, let (*V*_c_) denote its pre-collision velocity and (*n*) the outward unit normal to the wall. The post-collision velocity is then updated according to

28 *v⃑* = *v⃑*_c_ − 2(*n⃑*·*v⃑*_c_)*n⃑*

This boundary condition prevents particles from penetrating the solid wall and maintains numerical stability during particle tracking. However, it is not intended to represent the complete physical behaviour of particle–wall contact in the near-wall region. In a real system, near-wall behaviour may involve lubrication forces, van der Waals interactions, electrostatic repulsion, surface adhesion, rolling friction, and the retention or accumulation of magnetic particles near the wall. Because the present study focuses on the competition between lateral magnetophoretic migration and background advective transport within the bifurcation region, these complex near-wall interactions were not included in the principal model.

In summary, a one-way-coupled particle-tracking model was established by incorporating Stokes viscous drag, magnetophoretic driving force, and effective gravitational force. The model directly links the magnetic Ni component and particle structural parameters to the externally applied magnetic-field gradient, providing the theoretical basis for the subsequent investigation of magnetic-field-gradient-induced transitions in branch selection and the influence of Ni-layer structure on magnetically controlled transport performance.

### 2.5 Scope of applicability and model constraints

The numerical framework developed in this study is intended to quantify the coupled effects of background advection, magnetic-field-gradient actuation and Janus-particle structure on branch transport under controlled low-Reynolds-number conditions. To enable direct comparison among different operating conditions, a fixed idealised three-dimensional bifurcation geometry, prescribed inlet and outlet boundary conditions, and a consistent particle-release protocol were used throughout the simulations. The present geometry serves as a controlled microfluidic configuration rather than an anatomical reconstruction of a specific vascular bifurcation. Variations in daughter-branch diameter, bifurcation angle, three-dimensional curvature, local cross-sectional area and flow partitioning can modify the local velocity field, streamline topology, position of the flow separatrix and particle residence time. Accordingly, the quantitative transport efficiencies and threshold magnetic-field gradients reported here are specific to the present geometry and flow conditions. Application of the framework to other bifurcation geometries would require recalculation of the corresponding flow field and magnetic-guidance conditions.

Whole blood is represented in the present model as an incompressible Newtonian fluid with constant viscosity. This approximation provides a consistent baseline for isolating the competition between lateral magnetophoretic migration and background advection. The additional inlet-velocity analysis quantifies how changes in the strength of background advection affect branch guidance within the low-Reynolds-number regime, but it does not reproduce the full rheological complexity of physiological blood flow. In particular, shear-rate-dependent viscosity and temporal variations in flow rate may alter the local velocity distribution, viscous resistance and particle residence time, and may consequently shift the quantitative magnetic conditions required for branch selection. The present results are therefore interpreted primarily as mechanistic trends under controlled flow conditions.

For particle tracking, the Au/Ni–SiO_2_ Janus structure is represented by an equivalent rigid sphere having the same total volume and mass as the composite particle, whilst the actual Ni magnetic volume is retained when evaluating the magnetic response. This treatment incorporates the principal changes in particle mass, hydrodynamic size and magnetic-material content associated with variations in particle structure, while avoiding the additional complexity of explicitly resolving orientation-dependent hydrodynamics. Rotational motion, magnetic torque and orientation-dependent drag are not independently solved in the present model. Instead, the effective magnetic moment of the Ni layer is assumed, under a quasi-steady orientation approximation, to remain approximately aligned with the local total magnetic field, *m*‖*B*. The model therefore focuses on translational magnetophoretic transport. Orientation-dependent effects may nevertheless produce additional trajectory variations, particularly for particles travelling close to the streamline separatrix, where relatively small changes in lateral displacement can alter the final outlet allocation.

The magnetic-force formulation further employs a near-saturation, maximum-magnetic-response approximation for the Ni layer, with the saturation magnetisation taken as *M*_s_ = 4.90 × 10^5^ A m^−1^. The Helmholtz coils provide the bias magnetic field required to establish the prescribed field direction and magnetic response, whereas the Maxwell coils primarily generate the magnetic-field-magnitude gradient responsible for translational actuation. The magnetic-field strength, direction and ∇∣*B*∣ used in particle tracking are all evaluated consistently from the same reconstructed combined field rather than prescribed independently. As shown in Section 3.1, the magnetic flux density within the particle-accessible working region remains above the near-saturation criterion adopted in the present model over the investigated operating range. This comparison supports the internal consistency of the magnetic-response approximation used for the numerical calculations. Nevertheless, the field-dependent magnetisation of an experimentally fabricated Ni layer may differ from the adopted material model, and such differences may shift the quantitative magnetic-force and branch-guidance thresholds.

Magnetic-field gradients between 0.5 and 7.0 T m^−1^ are considered in the particle-transport simulations following the magnetic-field reconstruction and field-strength evaluation described above. This interval defines the operating range examined using the present equivalent three-axis Helmholtz–Maxwell coil model. The equivalent model is intended to establish the relationship between coil excitation and the magnetic conditions within the working region rather than to describe all electrical and thermal characteristics of a physical coil assembly. In an experimental actuation platform, the practically attainable magnetic-field gradient will additionally depend on parameters such as coil geometry, number of turns, conductor dimensions, driving-current capacity, working distance, effective actuation volume, power dissipation and allowable temperature rise. During sustained high-current operation, Joule heating may require active thermal management, such as forced-air or water cooling, depending on the specific coil configuration and operating conditions. These engineering factors would need to be evaluated when translating the reconstructed magnetic conditions to a physical electromagnetic actuation platform.

Changes in Ni-layer thickness are also treated as coupled structural variations rather than as changes in an isolated magnetic parameter. Increasing the Ni-layer thickness simultaneously modifies the magnetic-material volume, total particle volume, mass, effective density and equivalent hydrodynamic size and therefore influences magnetic driving, viscous resistance and gravitational effects. The Ni-layer thickness and Ni volume fraction should consequently be interpreted as descriptors of the composite particle structure. The mechanistic analysis in Section 3.5 compares the relative changes in magnetic response and hydrodynamic resistance to identify their contributions to the observed transport behaviour. The investigated Ni-layer thickness range is comparable to dimensions reported for experimentally fabricated magnetic Janus particles and microrobots,^20,27,28^ although practical implementation must also consider fabrication tolerances, overall particle dimensions and confinement within the transport channel. Within the parameter range examined here, increasing the magnetic-material content reduces the external magnetic-field gradient required to achieve a comparable branch-guidance performance, indicating that joint selection of particle magnetic structure and external actuation conditions can be useful in the design of future experimental systems.

Overall, the present framework is intended to identify mechanistic relationships among particle structure, background advection and magnetic actuation under controlled bifurcating-flow conditions. The quantitative thresholds depend on the specific geometrical, rheological, magnetic and particle-release conditions adopted here, whereas the principal outcome of the analysis is the identification of the relative trends and competing physical mechanisms governing branch transport.

## 3 Results and discussion

### 3.1 Magnetic-field-gradient reconstruction performance

Before applying the saturation-magnetisation approximation in the particle-tracking model, the reconstructed electromagnetic field was quantitatively evaluated to verify the controllability of the magnetic-field gradient and the validity of the assumed magnetic response of the Ni layer. In the present framework, the magnetic field was not directly prescribed as an ideal gradient field. Instead, the total magnetic field generated by the combined Helmholtz–Maxwell coil system was reconstructed through the linear superposition of individual coil responses, and the local magnetic-field-gradient magnitude was subsequently calculated from the reconstructed total field.

The accuracy of the reconstructed magnetic-field gradient was first examined over the range of 0.5–7 T m^−1^. As shown in Fig. 4(a), the mean reconstructed gradients exhibited excellent agreement with the prescribed target values over the entire investigated range. The reconstructed results remained almost identical to the ideal agreement line (*y* = *x*), indicating that the proposed coil-current optimisation strategy provides accurate regulation of the magnetic-field-gradient magnitude. For example, the reconstructed gradients at target values of 0.5 and 7 T m^−1^ were 0.5000 and 7.0000 T m^−1^, respectively, demonstrating the high linearity and accuracy of the reconstruction approach. The corresponding reconstructed gradient data were obtained from the mean values within the particle-accessible working region.

**Fig. 4:**
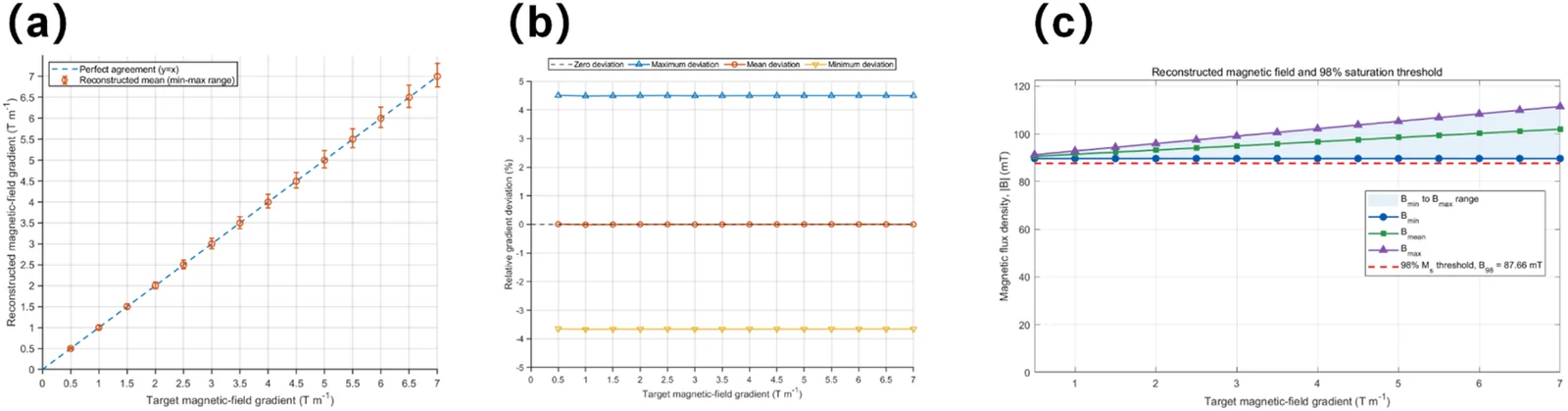
Validation of the reconstructed electromagnetic field. (a) Comparison between prescribed and reconstructed magnetic-field gradients. Error bars represent the minimum-to-maximum gradient range within the particle-accessible working region. (b) Relative deviations of the maximum, mean, and minimum reconstructed gradients from the prescribed values. (c) Reconstructed magnetic flux density within the working region and comparison with the Ni near-saturation threshold.

To further evaluate the spatial stability of the reconstructed gradient field, the maximum and minimum gradient values within the working region were compared with the mean reconstructed gradient. As presented in Fig. 4(b), the relative deviations of the reconstructed gradients remained close to zero for the mean values, while the deviations of the maximum and minimum gradients remained within a narrow range throughout the investigated conditions. The maximum and minimum reconstructed gradients increased proportionally with the target gradient, indicating that increasing the magnetic-gradient magnitude mainly scales the overall field intensity without significantly changing the spatial distribution characteristics of the reconstructed gradient field. The maximum and minimum gradient values used for this evaluation were obtained directly from the reconstructed field distribution within the working region.

The magnetic-field magnitude within the working region was further examined to ensure that the reconstructed electromagnetic conditions were sufficient for applying the saturation-magnetisation approximation in the particle-tracking model. As shown in Fig. 4(c), the minimum, mean, and maximum magnetic flux densities were evaluated under different reconstructed gradient conditions. Throughout the investigated operating range, the minimum magnetic-field magnitude within the particle-accessible region remained above the near-saturation threshold of Ni. This result indicates that the Ni magnetic layer can maintain a near-saturated magnetic state during particle transport under the reconstructed magnetic-field conditions.

### 3.2 Effect of magnetic-field gradient on branch guidance

After confirming that an oblique magnetic-field gradient directed towards the target branch could be stably reconstructed within the local working region, the effect of magnetic-field-gradient magnitude on the branch-transport behaviour of Au/Ni–SiO_2_ Janus microrobots was investigated. All simulations employed identical channel geometry, inlet velocity, outlet boundary conditions, and particle-release protocols, with only the applied magnetic-field gradient varied to ensure comparability between cases.

As shown in Fig. 5(a), a stable laminar velocity field was established within the dual-inlet–dual-outlet bifurcating model. In the absence of an externally applied magnetic-field gradient, the particles were transported predominantly along the background streamlines towards the downstream region. Janus microrobots released from inlet 2 were unable to generate sufficient active lateral displacement to cross the streamlines towards target outlet 1. Under these conditions, particle outlet selection was therefore governed primarily by the initial streamline position and background advection. The Au/Ni–SiO_2_ Janus microrobot with a Ni-layer thickness of 120 nm was first selected as the baseline structure to investigate the influence of magnetic-field gradient on particle branch selection. A total of 150 particles were tracked for each magnetic-field-gradient condition, and outlet counters were used to record separately the numbers entering target outlet 1 and non-target outlet 2. The 150 particles were released in three batches of 50 particles to facilitate trajectory management and outlet counting. Because the simulations employed a dilute-particle assumption and one-way coupling, no explicit interactions occurred between individual particles; consequently, the release batch itself was not treated as a physical parameter affecting particle dynamics.

**Fig. 5:**
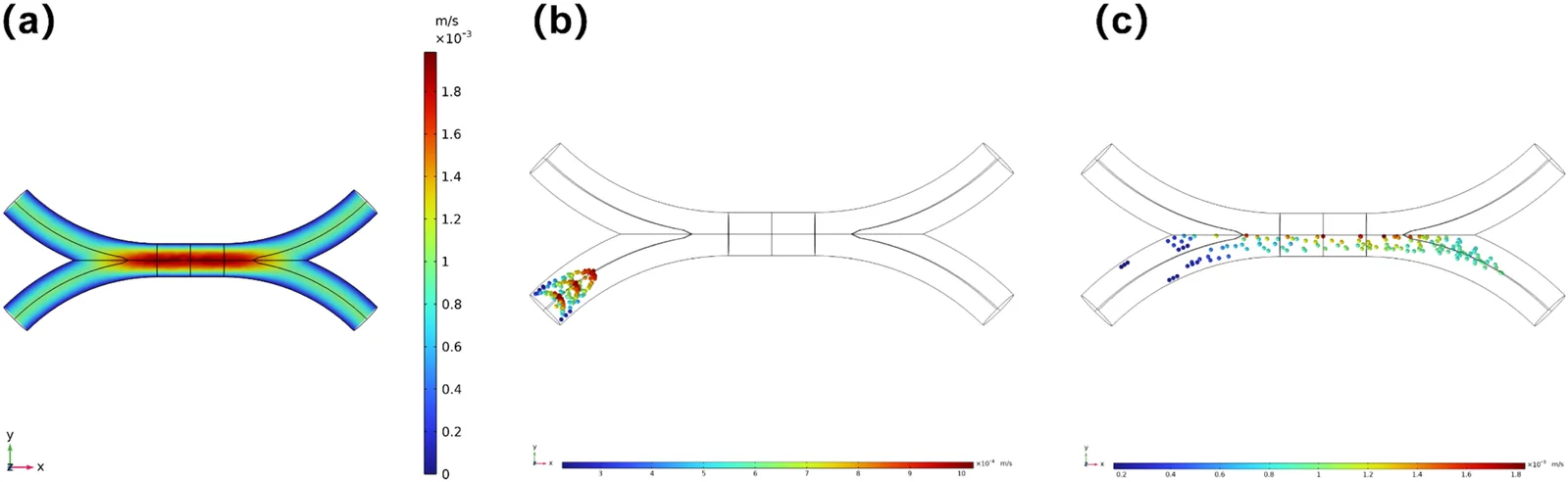
Background flow field and particle transport behaviour of the baseline Au/Ni–SiO_2_ Janus microrobots in the bifurcating microchannel under an inlet velocity of 0.5 mm s^−1^. (a) Steady-state velocity field of the bifurcating microchannel; (b) particle release at inlet 2; and (c) representative particle trajectories within the bifurcation region.

As shown in Fig. 6(a), the target-branch transport efficiency exhibited an overall increasing trend with increasing magnetic-field gradient. At a magnetic-field gradient of 1.5 T m^−1^, 64 particles entered the target branch, corresponding to a transport efficiency of 42.67%. When the gradient was increased to 3.0 T m^−1^, the number of particles entering the target branch increased to 88, giving a transport efficiency of 58.67%. With further increases in the magnetic-field gradient, deflection of the particles towards the target branch became progressively stronger. At 5 T m^−1^, the target-branch transport efficiency reached 86.67%.

**Fig. 6:**
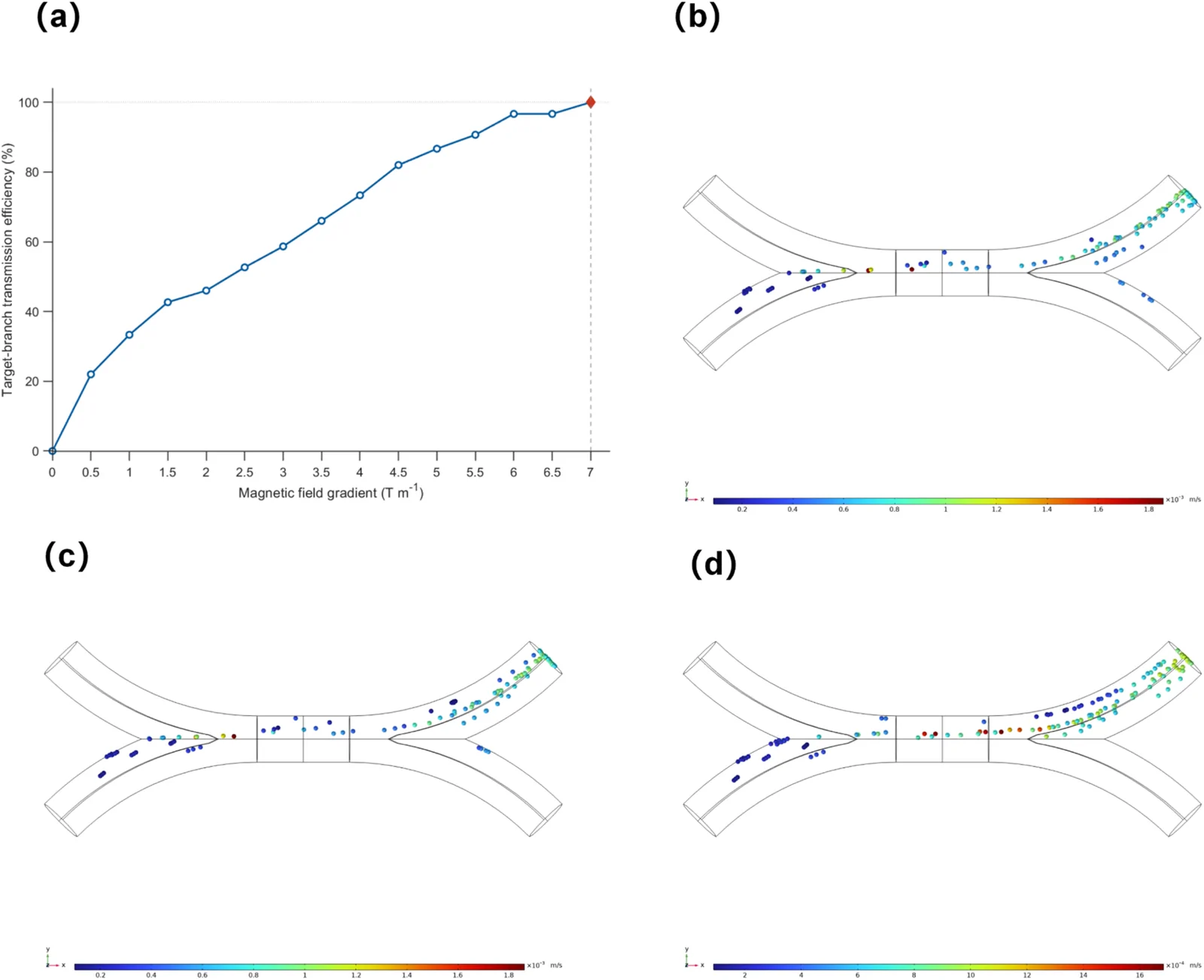
Effect of magnetic-field gradient on the branch-guidance performance of Au/Ni–SiO_2_ Janus microrobots. (a) Target-branch transport efficiency as a function of the applied magnetic-field gradient under an inlet velocity of 0.5 mm s^−1^ (b) 6 T m^−1^, 96.67%; (c) 6.5 T m^−1^, 96.67% (d) 7 T m^−1^, 100%.

When the magnetic-field gradient was further increased to 7.0 T m^−1^, all 150 particles entered the target branch, corresponding to a target-branch transport efficiency of 100%. Accordingly, 7.0 T m^−1^ was the minimum tested magnetic-field gradient at which 100% target-branch transport was first obtained under the present discrete test conditions.

Notably, at magnetic-field gradients of 6.0 and 6.5 T m^−1^, the target-branch transport efficiency remained at 96.67%, with 145 of the 150 tracked microrobots entering the target branch. This result indicates a short plateau in the transport efficiency over these two adjacent gradient conditions, despite the further increase in magnetic driving. Such behaviour does not imply that increasing the magnetic-field gradient produces no further change in particle motion; rather, it is consistent with a threshold-like response associated with magnetically controlled transport through the bifurcation.

As shown in Fig. 6(b) and (c), increasing the magnetic-field gradient continuously enhances the magnetophoretic force acting on the Ni-containing Janus microrobots, thereby increasing their lateral migration velocity and accumulated lateral displacement. However, branch selection is not determined solely by the magnitude of the magnetic force, but by whether the lateral displacement accumulated within the finite residence time is sufficient for the particles to cross the streamline separatrix governing downstream outlet allocation. At 6.0 and 6.5 T m^−1^, although the stronger magnetic driving further promotes lateral migration, five particles remain unable to cross the relevant separatrix and therefore enter the non-target branch. Consequently, the target-branch transport efficiency remains unchanged at 96.67%.

When the magnetic-field gradient is further increased to 7.0 T m^−1^ [Fig. 6(d)], the enhanced magnetophoretic migration provides sufficient additional lateral displacement for the particles that were previously close to the branch-selection threshold to cross the effective streamline separatrix and enter the target branch. As a result, all 150 tracked microrobots are directed into the target branch, corresponding to a target-branch transport efficiency of 100%. These results suggest that, under the present simulation conditions, the transition from high-efficiency transport to complete target-branch guidance occurs within the interval between the two adjacent tested gradients of 6.5 and 7.0 T m^−1^.

Thus, the macroscopic increase in target-branch transport efficiency reflects an enhancement of lateral magnetophoretic migration relative to axial background advective transport. Under the flow conditions, particle structure, and magnetic-field orientation considered here, increasing the applied magnetic-field gradient therefore markedly enhanced lateral magnetophoretic migration of Au/Ni–SiO_2_ Janus microrobots within the bifurcation region and increased the proportion of particles entering the target branch. These numerical results support the feasibility of controlling the branch guidance of Janus microrobots by adjusting the externally applied magnetic-field gradient.

### 3.3 Effect of inlet flow velocity on branch-guidance performance

To further investigate the influence of background advection on magnetically controlled branch guidance, the inlet velocity was varied from 0.25 to 1.50 mm s^−1^, whilst the structure and magnetic properties of the baseline Janus microrobot, the particle-release conditions, and all other boundary conditions were kept unchanged. The corresponding Reynolds numbers are first evaluated in Fig. 7(a). At inlet velocities of 0.25, 0.50, 1.00, and 1.50 mm s^−1^, the Reynolds numbers are approximately 0.066, 0.133, 0.265, and 0.398, respectively, and therefore remain below 0.4 throughout the investigated range. This confirms that all cases considered remain within the low-Reynolds-number regime, where viscous effects dominate.

**Fig. 7:**
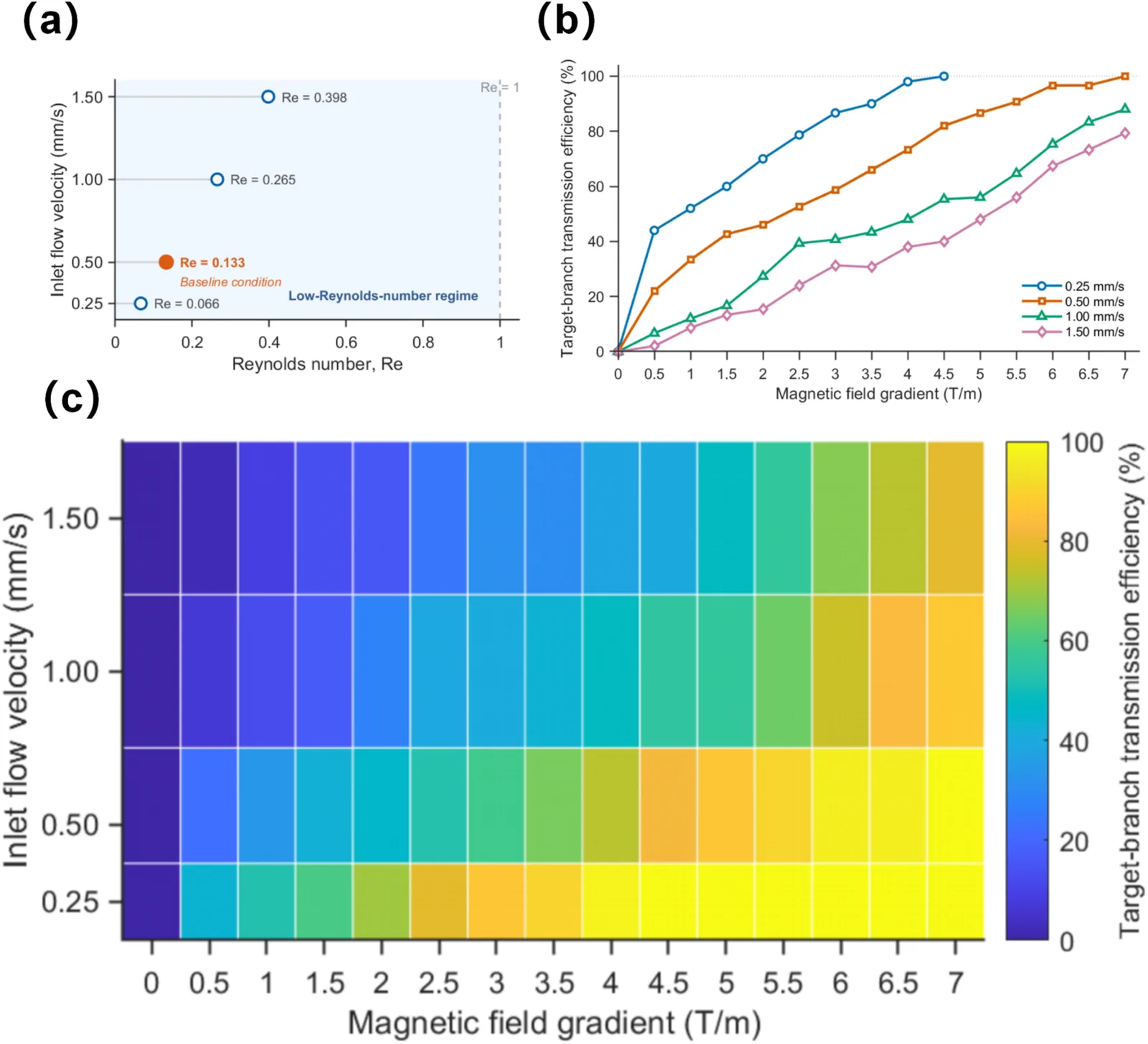
Effect of inlet flow velocity on the magnetic branch-guidance performance of Au/Ni–SiO_2_ Janus microrobots. (a) Reynolds numbers corresponding to inlet velocities of 0.25, 0.50, 1.00, and 1.50 mm s^−1^, confirming that all investigated conditions remain within the low-Reynolds-number regime. (b) Target-branch transport efficiency as a function of magnetic-field gradient at different inlet velocities. (c) Magnetic-field-gradient–inlet-velocity performance map of the target-branch transport efficiency.

Fig. 7(b) shows the target-branch transport efficiency as a function of magnetic-field gradient at different inlet velocities. Overall, increasing the magnetic-field gradient enhances transport towards the target branch, whereas increasing the inlet velocity shifts the response curves towards higher magnetic-field gradients. This indicates that stronger background advection requires correspondingly stronger magnetophoretic driving to maintain a comparable level of branch guidance. For example, at a magnetic-field gradient of 4 T m^−1^, the target-branch transport efficiencies are 98.0%, 73.3%, 48.0%, and 38.0% at inlet velocities of 0.25, 0.50, 1.00, and 1.50 mm s^−1^, respectively. Thus, under the same magnetic driving condition, increasing the background flow markedly reduces the proportion of particles transported into the target branch.

The inlet velocity also has a pronounced effect on the magnetic-field gradient required to achieve complete target-branch transport. At 0.25 mm s^−1^, a target-branch transport efficiency of 100% is first obtained at 4.5 T m^−1^ among the discrete gradients tested. By comparison, under the baseline inlet velocity of 0.50 mm s^−1^, complete target-branch transport is first achieved at 7 T m^−1^. When the inlet velocity is further increased to 1.00 and 1.50 mm s^−1^, the corresponding efficiencies at the maximum tested gradient of 7 T m^−1^ are 88.0% and 79.3%, respectively, and complete branch guidance is not reached within the present gradient range. These results show that, under the present simulation conditions, the magnetic driving required to achieve a comparable level of branch guidance generally increases with inlet velocity.

This flow-velocity dependence can be understood from the competition between lateral magnetophoretic migration and axial background advection. For a given magnetic-field gradient, reducing the inlet velocity increases the residence time of the particles within the effective magnetic-actuation region, allowing the magnetophoretic lateral velocity to accumulate into a greater lateral displacement. The particles are therefore more likely to cross the streamline separatrix governing downstream outlet allocation before reaching the bifurcation. Conversely, increasing the inlet velocity strengthens axial advection and shortens the effective magnetic-actuation time, thereby reducing the accumulated lateral displacement at a given magnetic-field gradient. A larger magnetic-field gradient is consequently required to compensate for the enhanced background advection. The relative displacement of the response curves in Fig. 7(b) therefore reflects a change in the balance between the characteristic effects of magnetophoretic migration and advective transport.

Fig. 7(c) further summarises the coupled effects of inlet velocity and magnetic-field gradient in a two-dimensional performance map. Each cell represents an explicitly simulated condition, without interpolation, and therefore directly reflects the results of the discrete parameter sweep. High target-branch transport efficiencies are concentrated in the low-inlet-velocity/high-magnetic-field-gradient region, whilst the high-efficiency region progressively shifts towards larger magnetic-field gradients as the inlet velocity increases. At 0.25 mm s^−1^, a continuous region of 100% target-branch transport is obtained over 4.5–7 T m^−1^. At 0.50 mm s^−1^, complete transport is achieved only at 7 T m^−1^, whereas no 100% transport region is obtained for inlet velocities of 1.00 and 1.50 mm s^−1^ within the investigated range of 0–7 T m^−1^ The performance map therefore reveals a clear compensatory relationship between background flow and magnetic driving: as axial advection increases, a larger magnetic-field gradient is required to maintain a comparable branch-guidance performance.

Taken together, Fig. 7 characterises the effect of inlet velocity from three complementary perspectives: validation of the flow regime, quantitative branch-transport response, and the coupled parameter space. Within the investigated low-Reynolds-number regime, target-branch transport is governed by the relative competition between lateral magnetophoretic migration and axial background advection. Increasing the inlet velocity does not alter the overall low-Reynolds-number flow regime considered here, but it increases the magnetic-field-gradient requirement for achieving high-efficiency or complete target-branch guidance.

### 3.4 Effect of Ni-Layer structure on magnetic transport

Having established the effect of magnetic-field gradient on the branch-transport behaviour of the baseline Au/Ni–SiO_2_ Janus microrobot, the influence of the Ni-layer structural parameters on the magnetic component of the particle and its magnetically controlled guidance performance was subsequently investigated. The relationship between the Ni-layer thickness, and the magnetic Ni volume fraction was first examined. As shown in Fig. 8(a), with the SiO_2_-core size and Au-layer thickness held constant, increasing (*t*_Ni_) from 150 to 390 nm resulted in a monotonic increase in (*ϕ*_Ni_) from 8.453% to 20.744%. This result indicates that, within the geometric range considered, increasing the Ni-layer thickness provides continuous control over the relative amount of magnetic material within the composite particle.

**Fig. 8:**
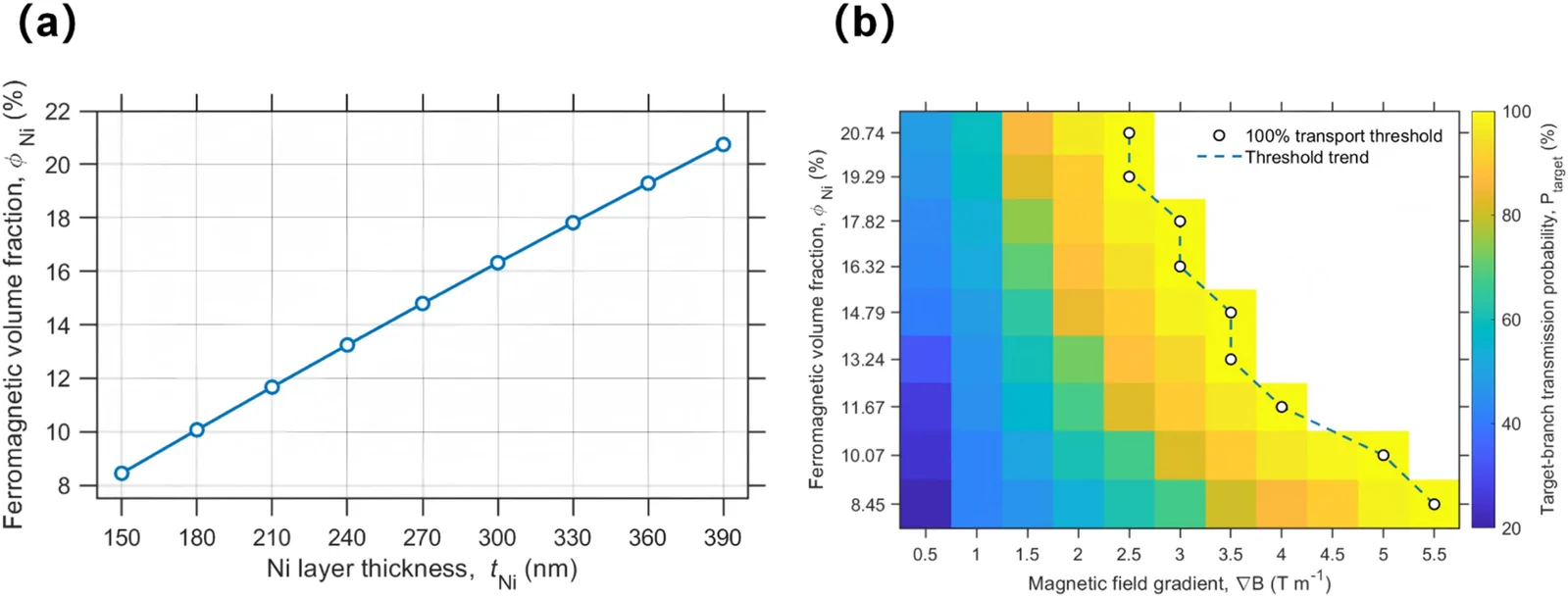
Effect of Ni-layer structure and magnetic-field gradient on the target-branch transport performance of Au/Ni–SiO_2_ Janus microrobots. (a) Variation of the magnetic Ni volume fraction, *ϕ*_Ni_, with Ni-layer thickness, with the SiO_2_-core size and Au-layer thickness held constant. (b) Target-branch transport efficiency as a function of *ϕ*_Ni_ and magnetic-field gradient, ∇∣*B*∣. The colour scale represents the target-branch transport efficiency. Open circles indicate, for each *ϕ*_Ni_, the minimum tested magnetic-field gradient at which 100% target-branch transport was first achieved, and the dashed line indicates the corresponding trend. Blank regions indicate conditions that were not further simulated after complete target-branch transport had been achieved.

The particle-tracking results were then used to analyse the combined effects of (*ϕ*_Ni_) and the externally applied magnetic-field gradient on the target-branch transport efficiency. As shown in Fig. 8(b), at a given magnetic-field gradient, the target-branch transport efficiency generally increased with increasing (*ϕ*_Ni_). Correspondingly, the minimum tested magnetic-field gradient required to achieve 100% target-branch transport decreased progressively. When (*ϕ*_Ni_) increased from 8.45% to 20.74%, the magnetic-field gradient at which 100% transport was first observed in the present discrete parameter sweep decreased from 5.5 to 2.5 T m^−1^, corresponding to a reduction of approximately 54.5%.The numerical results therefore indicate that increasing the Ni-layer thickness can substantially reduce the external magnetic-field gradient required to achieve complete transport to the target branch under the conditions considered.

Fig. 9 further presents representative particle trajectories corresponding to the first occurrence of 100% target-branch transport for different values of (*ϕ*_Ni_).Although all of these cases ultimately resulted in complete transport of the particles into the target branch, the magnetic-field gradient required to achieve the same transport outcome generally decreased as (*ϕ*_Ni_) increased, consistent with the parametric-sweep results shown in Fig. 8. This behaviour indicates that the Ni-layer structure can effectively regulate the response of the Janus microrobot to an externally applied non-uniform magnetic field and thereby modify the magnetic actuation conditions required for branch guidance. Parameter sweeps performed for different Ni-layer thicknesses show that increasing the Ni-layer thickness generally improves the target-branch transport efficiency at a given magnetic-field gradient and reduces the external magnetic-field gradient required to achieve complete target-branch transport. As the Ni volume fraction increased from 8.45% to 20.74%, the minimum tested magnetic-field gradient at which 100% target-branch transport was first achieved decreased from 5.5 to 2.5 T m^−1^ within the present discrete parameter sweep. Meanwhile, local plateaux in the minimum tested gradient were observed for adjacent Ni-layer thicknesses: 240–270 nm, 300–330 nm, and 360–390 nm corresponded to 3.5, 3.0, and 2.5 T m^−1^, respectively. These plateaux do not imply that further increases in Ni-layer thickness produce no additional change in the particle dynamics. Rather, with a discrete magnetic-field-gradient increment of 0.5 T m^−1^ and a 100% outlet-allocation criterion, the additional enhancement in magnetophoretic migration is not necessarily sufficient to shift the observed threshold to the next lower tested gradient. Overall, these results indicate a degree of compensation between the Ni magnetic-layer structure and the externally applied magnetic-field gradient: increasing the magnetic-material content can, within the investigated parameter range, reduce the external magnetic driving required to achieve a comparable level of branch-guidance performance.

**Fig. 9:**
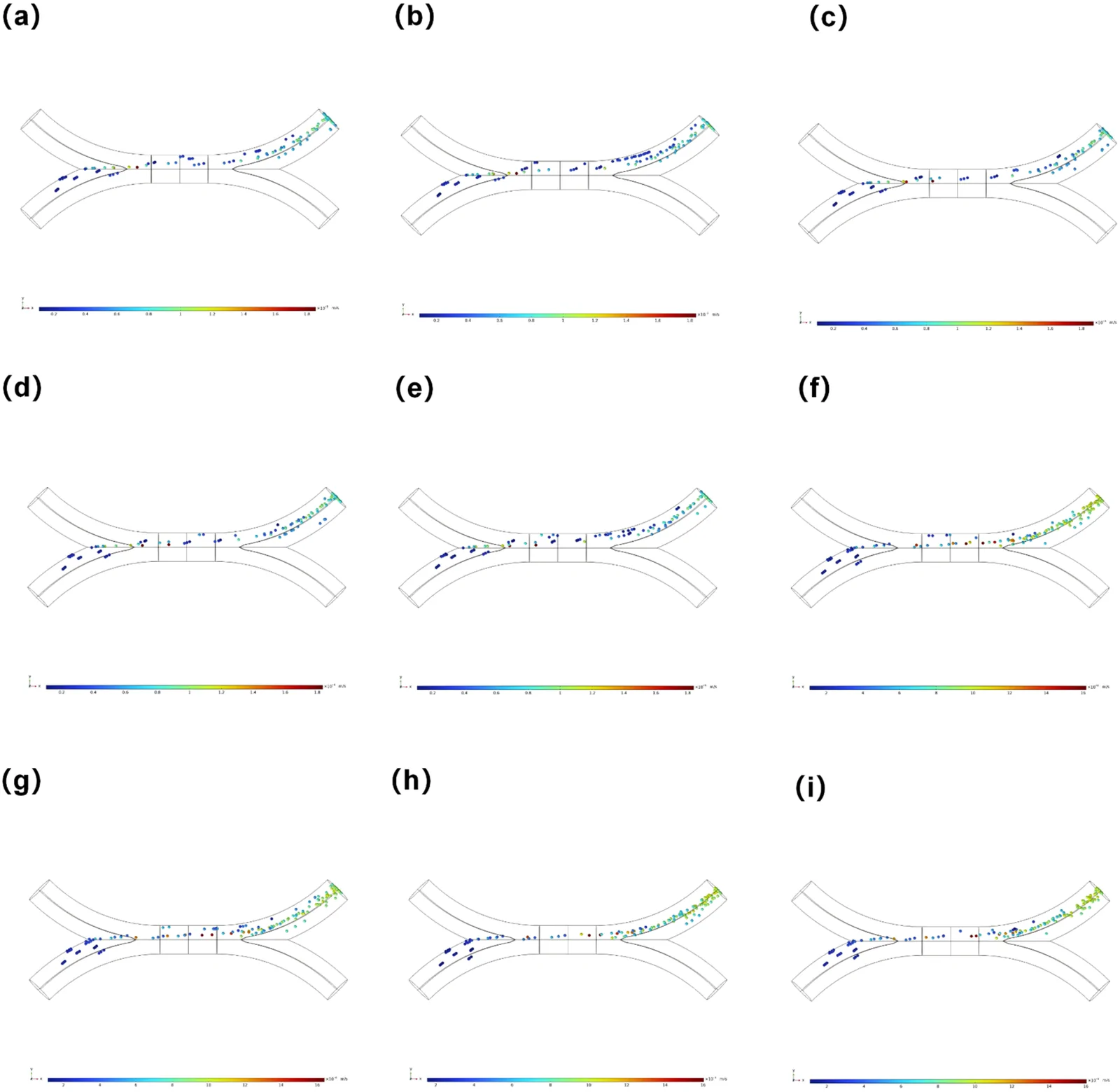
Representative particle trajectories achieving 100% target-branch transport at different Ni volume fractions. (a) *ϕ*_Ni_ = 8.45%,∇∣*B*∣ = 5.5 T m^−1^ (b) *ϕ*_Ni_ = 10.07%,∇∣*B*∣ = 5 T m^−1^ (c) *ϕ*_Ni_ = 11.67%,∇∣*B*∣ = 4 T m^−1^ (d) *ϕ*_Ni_ = 13.24%, ∇∣*B*∣ = 3.5 T m^−1^ (e) *ϕ*_Ni_ = 14.79%, ∇∣*B*∣ = 3.5 T m^−1^ (f) *ϕ*_Ni_ = 16.32%, ∇∣*B*∣ = 3 T m^−1^ (g) *ϕ*_Ni_ = 17.82%, ∇∣*B*∣ = 3 T m^−1^ (h) *ϕ*_Ni_ = 19.29%, ∇∣*B*∣ = 2.5 T m^−1^ (i) *ϕ*_Ni_ = 20.74%, ∇∣*B*∣ = 2.5 T m^−1^.

Within the range of Au/Ni–SiO_2_ structural parameters investigated here, increasing the Ni-layer thickness therefore generally enhanced magnetically induced lateral deflection and reduced the externally applied magnetic-field gradient required to achieve complete target-branch transport. In practical structural design, however, increasing the Ni-layer thickness must be considered together with the associated enhancement in magnetic response, increase in overall particle size, and geometric constraints on passage through the channel. Accordingly, provided that fabrication requirements and geometric transport constraints are satisfied, co-optimisation of the magnetic-material content and external magnetic-field gradient represents an important design strategy for reducing magnetic actuation requirements and improving branch-guidance efficiency.

### 3.5 Effect of Ni-Layer structure on magnetic transport

To further clarify the relative contributions of the physical parameters altered by increasing the Ni-layer thickness, the magnetic moment, particle mass, effective density, and hydrodynamic resistance were compared over the Ni-layer-thickness range of 120–390 nm. Fig. 10(a) presents these parameters normalised to the values for the 120 nm Ni-layer case. As the Ni-layer thickness increased from 120 to 390 nm, the particle magnetic moment increased from 2.422 × 10^−12^ to 8.736 × 10^−12^ A m^2^, corresponding to approximately 3.61 times the baseline value. By comparison, the particle mass and effective density increased to approximately 1.54 and 1.30 times their baseline values, respectively. The equivalent hydrodynamic radius increased only from 2.588 to 2.738 µm, resulting in an increase of approximately 5.8% in the Stokes drag coefficient, which scales directly with *R*_h_. These results show that, within the investigated structural range, the increase in magnetic moment associated with increasing Ni-layer thickness is substantially greater than the accompanying increase in hydrodynamic resistance, indicating a marked enhancement of the magnetic response relative to viscous resistance. To further examine whether these parameter variations can account for the transport behaviour observed at different Ni-layer thicknesses, all particle-tracking results were re-expressed in terms of the dimensionless magnetic-guidance number, *Λ*_m_ defined as:29
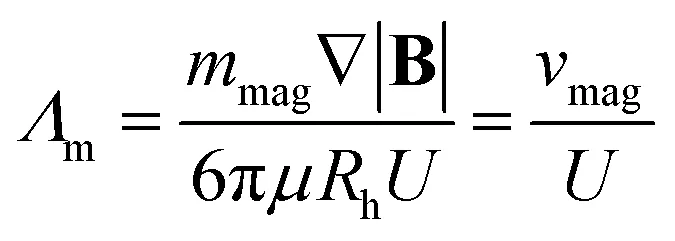


**Fig. 10:**
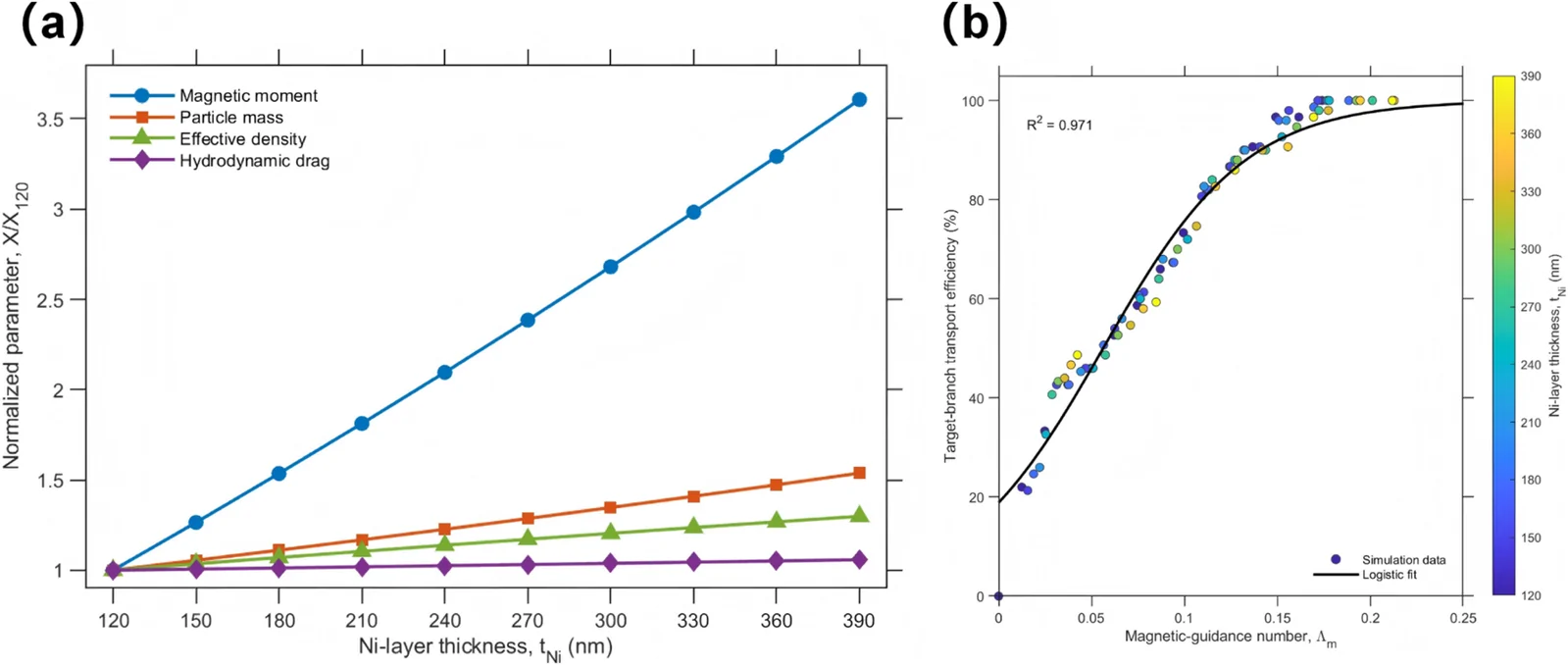
Effects of Ni-layer thickness on the magnetic response, hydrodynamic properties, and branch-transport performance of Au/Ni–SiO_2_ Janus microrobots. (a) Normalised variations in magnetic moment, particle mass, effective density, and Stokes drag coefficient with Ni-layer thickness, using the 120 nm Ni-layer case as the reference. (b) Target-branch transport efficiency as a function of the dimensionless magnetic-guidance number, *Λ*_m_, for different Ni-layer thicknesses.

As shown in Fig. 10(b), the original particle-tracking simulations comprise ten Ni-layer thicknesses ranging from 120 to 390 nm, with different magnetic-field gradients and corresponding transport efficiencies. When these results are expressed as a function of *Λ*_m_, the data for the different Ni-layer thicknesses largely collapse onto a common sigmoidal response. An empirical logistic fit to all simulation data gives *R*^2^ = 0.971, an RMSE of 0.042. Although different Ni-layer thicknesses are also associated with changes in particle mass, density, and hydrodynamic size, these coupled changes are retained in the full particle-tracking simulations from which the transport efficiencies were obtained. The resulting data collapse when expressed in terms of *Λ*_m_ therefore indicates that the target-branch transport performance is governed primarily by the relative competition between magnetophoretic driving, viscous resistance, and background advection, rather than by Ni-layer thickness acting as an independent geometric control parameter.

Taken together, Fig. 10 shows that increasing the Ni-layer thickness simultaneously alters the particle magnetic moment, mass, effective density, and hydrodynamic size, but these parameters change to markedly different extents. Over the range of 120–390 nm, the magnetic moment increases by approximately 260.7%, whereas the Stokes drag coefficient increases by only about 5.8%. In addition, the transport results for different Ni-layer thicknesses can be represented consistently using the dimensionless parameter *Λ*_m_, which incorporates both magnetic moment and hydrodynamic resistance. Therefore, within the investigated parameter range and modelling conditions, the pronounced enhancement of magnetic response relative to hydrodynamic resistance is the principal contribution to the improved branch-guidance performance and the reduced external magnetic-field-gradient requirement associated with increasing Ni-layer thickness. Changes in particle mass, effective density, and other structural parameters remain incorporated in the full particle dynamics and should not be regarded as negligible; however, the present results indicate that they are not the primary factors governing the overall performance trend.

## 4 Conclusions

A three-dimensional numerical framework was developed to investigate the magnetic-field-gradient-guided transport of Au/Ni–SiO_2_ Janus microrobots in an idealised low-Reynolds-number bifurcating microchannel. An equivalent three-axis Helmholtz–Maxwell coil model was used to reconstruct an oblique magnetic-field gradient directed towards the target branch. Over the investigated range of 0.5–7.0 T m^−1^, the reconstructed gradients closely matched the prescribed values, while the magnetic flux density within the particle-accessible region remained above the adopted near-saturation criterion for Ni, providing consistent magnetic conditions for particle-transport simulations.

For the baseline microrobot with a 120 nm Ni layer, increasing the magnetic-field gradient enhanced lateral magnetophoretic migration and increased the target-branch transport efficiency from 42.67% at 1.5 T m^−1^ to 100% at 7.0 T m^−1^. A short efficiency plateau of 96.67% was observed at 6.0–6.5 T m^−1^, reflecting the threshold-like nature of branch allocation in bifurcating flow. Increasing the inlet velocity from 0.25 to 1.50 mm s^−1^ reduced the available magnetic-actuation time and shifted the high-efficiency guidance region towards higher magnetic-field gradients, confirming the competition between magnetophoretic migration and background advection.

Increasing the Ni-layer thickness from 150 to 390 nm raised the magnetic Ni volume fraction from 8.45% to 20.74% and reduced the minimum tested gradient required for complete target-branch transport from 5.5 to 2.5 T m^−1^, a reduction of approximately 54.5%. Over the broader thickness range of 120–390 nm, the magnetic moment increased by approximately 260.7%, whereas the Stokes drag coefficient increased by only approximately 5.8%. Transport data for different Ni-layer thicknesses could also be largely represented by a common response using a dimensionless magnetic-guidance parameter. These results indicate that the improved guidance performance is mainly governed by the enhancement of magnetic driving relative to hydrodynamic resistance and background advection.

Overall, branch-selection performance is jointly determined by particle magnetic structure, background flow velocity and external magnetic-field gradient. Their coupled optimisation can therefore reduce the magnetic actuation required for high-efficiency branch guidance. The quantitative thresholds reported here are specific to the present idealised geometry, flow conditions, particle-release protocol and magnetic-response assumptions, and may vary under different operating conditions. In addition, the equivalent Helmholtz–Maxwell coil model represents the magnetic response of the actuation system rather than its complete electrical and thermal performance. Practical implementation would require further consideration of coil geometry, driving current, power dissipation and thermal management, including active cooling where necessary. The present framework provides a quantitative basis for the coupled design of magnetic particle structure and external actuation and can be extended to more realistic bifurcation geometries, flow rheology, particle orientational dynamics, experimentally characterised actuation systems and experimental validation.

## Author contributions

Junbo Yang: formal analysis (lead), data curation (lead), methodology (lead), validation (lead), visualization (lead), writing – original draft (lead), writing – review & editing (supporting). Meijuan Li: conceptualization (supporting), methodology (supporting), writing – review & editing (supporting). Yonghui Yang: resources (supporting), writing – review & editing (supporting). Xue-bo Chen: supervision (lead), writing – review & editing (lead).

## Conflicts of interest

There are no conflicts to declare.

## Supplementary Material

RA-OLF-D6RA08407A-s001

RA-OLF-D6RA08407A-s002

## Data Availability

The data supporting the findings of this study are provided within the article and its supplementary information (SI). Supplementary information: magnetic-field reconstruction data, target-branch transport data for the baseline Janus microrobot with a 120 nm Ni layer, and target-branch transport data for Janus microrobots with different Ni-layer thicknesses. See DOI: https://doi.org/10.1039/d6ra08407a.
